# All-Trans Retinoic Acid Modulates TLR4/NF-*κ*B Signaling Pathway Targeting TNF-*α* and Nitric Oxide Synthase 2 Expression in Colonic Mucosa during Ulcerative Colitis and Colitis Associated Cancer

**DOI:** 10.1155/2017/7353252

**Published:** 2017-03-20

**Authors:** Hayet Rafa, Sarra Benkhelifa, Sonia AitYounes, Houria Saoula, Said Belhadef, Mourad Belkhelfa, Aziza Boukercha, Ryma Toumi, Imene Soufli, Olivier Moralès, Yvan de Launoit, Hassen Mahfouf, M'hamed Nakmouche, Nadira Delhem, Chafia Touil-Boukoffa

**Affiliations:** ^1^Team: Cytokines and NO Synthases-Immunity and Pathogenesis, Laboratory of Cellular and Molecular Biology (LBCM), Faculty of Biological Science, University of Sciences and Technology (USTHB), Algiers, Algeria; ^2^Institut de Biologie de Lille, UMR 8161, CNRS, Institut Pasteur de Lille, Université Lille-Nord de France, Lille, France; ^3^Anatomic Pathology Service, Mustapha Pacha Hospital, Algiers, Algeria; ^4^Department of Gastroenterology, Maillot Hospital, Algiers, Algeria; ^5^Service of Oncology, Rouiba Hospital, Algiers, Algeria

## Abstract

Colitis associated cancer (CAC) is the colorectal cancer (CRC) subtype that is associated with bowel disease such as ulcerative colitis (UC). The data on role of NF-*κ*B signaling in development and progression of CAC were derived from preclinical studies, whereas data from human are rare. The aim of this work was to study the contribution of NF-*κ*B pathway during UC and CAC, as well as the immunomodulatory effect of all-trans retinoic acid (AtRA). We analyzed the expression of NOS2, TNF-*α*, TLR4, and NF-*κ*B, in colonic mucosa. We also studied NO/TNF-*α* modulation by LPS in colonic mucosa pretreated with AtRA. A marked increase in TLR4, NF-*κ*B, TNF-*α*, and NOS2 expression was reported in colonic mucosa. The relationship between LPS/TLR4 and TNF-*α*/NO production, as well as the role of NF-*κ*B signaling, was confirmed by ex vivo experiments and the role of LPS/TLR4 in NOS2/TNF-*α* induction through NF-*κ*B pathway was suggested. AtRA downregulates NOS2 and TNF-*α* expression. Collectively, our study indicates that AtRA modulates in situ LPS/TLR4/NF-*κ*B signaling pathway targeting NOS2 and TNF-*α* expression. Therefore, we suggest that AtRA has a potential value in new strategies to improve the current therapy, as well as in the clinical prevention of CAC development and progression.

## 1. Introduction

Colorectal cancer (CRC) is one of the most common lethal cancers worldwide [1]. There are two major types of CRC, sporadic colorectal carcinoma (SCC) and colitis associated cancer (CAC) [2]. Factors that may increase the risk of colorectal cancer have been extensively studied [3, 4]. Physical inactivity, obesity, smoking, and dietary patterns such as high red and processed meat consumption as well as moderate-to-heavy alcohol use also increase the risk for CRC [4–6]. Several reports have shown that chronic inflammation predisposes individuals to various types of cancer [3, 7, 8]. The risk of colorectal cancer is increased in patients with inflammatory bowel disease, particularly in long-standing and extensive ulcerative colitis (UC) [8]. Then, colitis associated cancer (CAC) is the CRC subtype that is associated with bowel disease (Crohn's disease (CD) and ulcerative colitis (UC)) [9]. Both diseases are characterized by the immune dysregulation in the intestine, involving a wide range of molecules leading to a chronically inflamed environment [10–12].

Although many reports have documented the critical link between inflammation and development of colon cancer, our knowledge of the underlying mechanisms remains incomplete. Different TLR ligands have been implicated in various experimental tumor models and are known to play different roles. While some TLRs contribute to antitumor responses, others conversely promote tumor growth and facilitate the evasion of immune surveillance [13]. TLR4 signaling plays a crucial role in the generation of innate response but also serves to activate the adaptive immune system in response to cancer [13, 14]. Activation of TLR4 by Gram-negative lipopolysaccharide (LPS) leads to the NF-*κ*B activation in the intestinal mucosa [15] and induced the expression of many proinflammatory molecules including cytokines and adhesion molecules [16, 17]. Among these cytokines, tumor necrosis factor-alpha (TNF-*α*) can further enhance NF-*κ*B activation in various cell types [18]. TNF-*α* is a multifunctional cytokine involved in apoptosis, cell survival, inflammation, and immunity acting via two receptors (TNF receptor p55 or TNF-Rp75) [19, 20].

Proinflammatory mediators and cytokines such as nitric oxide (NO) and TNF-*α* play important roles in regulating inflammatory response. Generation and secretion of a high NO concentration by infiltrating cells and resident activated macrophages may lead to perpetuation of local tissue damage [21, 22]. The inducible NOS (iNOS) is expressed following stimulation with lipopolysaccharide and/or inflammatory cytokines such as TNF-*α* [22–24]. The activation of iNOS leads to prolonged production of NO in high, potentially cytotoxic concentrations [25]. In our previous studies, we reported that chronic UC is characterized by overexpression of iNOS with high levels of NO generation by PBMC and colonic mucosa stimulated with IFN-*γ* or IL-17A and correlates with histological damage [10, 11]. Thus iNOS generated NO may give the cell a double hit by both damaging the DNA and inhibiting its repair processes. This effect of NO and its by-products may make NO one of the pivotal mediators linking inflammation to carcinogenesis [25]. These observations suggest that iNOS may also play a fundamental role in the enhancement of colon cancers risk in IBD patients, as well as in promotion/progression of cancers arising within a background of inflammation [26]. However, the data on role of TLR4/NF-*κ*B signaling in TNF-*α*/iNOS induction in UC-associated carcinogenesis are still not fully understood and derived from preclinical studies, whereas data from human CAC are rare.

Great progress has been made in the development of chemotherapy, as well as targeted therapies for advanced different forms of CRC. Meanwhile, many cases show that tolerance develops to such treatments [27]. However, the treatment of different forms of CCR included CAC requires new strategies to improve the current therapy. It has been reported that induction of NF-kB activation leads to the resistance to chemotherapy [28].

Accumulative researches indicated that retinoids were associated with the prevention and amelioration of numerous chronic diseases and cancers [29–32]. Retinoids are currently used as chemotherapies against cancers of epithelial origin [33]. All-trans retinoic acid (AtRA) is an active metabolite of retinoid and regulates a wide range of biologic processes through the action of two families of nuclear retinoid receptors, retinoid acid receptor (RAR) and retinoid X receptor (RXR). Heterodimers composed of an RAR and an RXR bind to specific RA response elements (RAREs) in target genes [34, 35].

AtRA has been shown to exert immunomodulatory and anti-inflammatory functions in various cell types [36–38]. In our previous studies, we reported that AtRA inhibited the NO production in proinflammatory cytokines stimulated peripheral blood mononuclear cells (PBMC) and monocytes from IBD and Alzheimer disease patients [11, 39]. Furthermore, retinoids are known to affect signaling pathways frequently altered which result in the development and progression of CRC [32]. In this sense, understanding the pathway involved in cancer-related inflammation and targeting transcription factors such as NF-*κ*B has attracted our attention. In this way, we investigate the contribution of TLR4/NF-*κ*B pathway in UC and CAC Algerian patients, as well as the effect of AtRA on TNF-*α* and NOS2 expression.

## 2. Patients and Methods

### 2.1. Patients

Thirty-five Algerian patients with ulcerative colitis, UC (14 men and 16 women; mean age 39.75 ± 9.23 years; range 22–53 years), and 9 patients with colitis associated cancer, CAC (3 men and 6 women; mean age 55.88 ± 6.66 years; range 48–67 years), were enrolled in this study. UC and CAC patients were diagnosed by standard endoscopic and histological examination in the Department of Gastroenterology, Maillot Hospital, Algiers, Algeria, Service of Oncology, Public hospital Rouiba Algiers Algeria and Anatomic Pathology service, Mustapha Pacha Algiers Algeria.

The histopathological examination confirmed the diagnosis of UC active stage and cancer. Healthy controls (*n* = 16) were obtained from adult volunteer donors. Each patient has given a written informal consent for the study required by the ethic committee of the national agency of research development in health (ATRSS) which supported our project.

### 2.2. Plasma Collection

Blood samples collected from healthy donors and patients were centrifuged at 2,000 rpm for 10 min to obtain plasma. All plasma samples were stored at −45°C until TNF-*α* and nitric oxide (NO) determination.

### 2.3. Colonic Biopsies and Culture

Multiple colonic biopsies were taken from patients who underwent colonoscopy in the Department of Gastroenterology, Maillot Hospital (Algiers, Algeria), Anatomic Pathology Service, Mustapha Pacha (Algiers, Algeria), and Service of Oncology, Public Hospital Rouiba, (Algiers, Algeria). Our study included biopsies from inflamed mucosa of patients with UC in active stage (*n* = 12) and with CAC (*n* = 6). Biopsies from normal mucosa (*n* = 6) were also used as negative controls. Colonic biopsies were immediately placed in the transport medium Hanks' balanced salts solution, pH 7.4, supplemented with antibiotics. Cultures of colonic mucosa were pretreated with all-trans retinoic acid (AtRA) at 10^-7 ^M for 6 h and stimulated with lipopolysaccharide (LPS; 10 *μ*g/mL). To confirm the engagement of NF-kB signaling pathway on TNF-*α* and NO production, colonic biopsies were stimulated with LPS (10 *μ*g/mL) and SN50, an inhibitor peptide of NF-*κ*B, AP-1, and STAT pathways (50 *μ*M/mL). The cultures were incubated at 37°C in an atmosphere of 5% CO_2_ up to 24 hours. Supernatants were then collected for NO (nitrite) and TNF-*α* measurement. The estimation of total protein per well was performed using Bradford method and nitrite or TNF-*α* contents of each well were expressed as *μ*M/mg or pg/mL of total protein, respectively [40].

### 2.4. Enzyme-Linked Immunosorbent Assay for Human TNF-*α*

Plasma TNF-*α* levels were determined by using enzyme-linked immunosorbent assay (ELISA) kit according to the manufacturer's instructions (Invitrogen-Life Technologies, USA). The absorbance was read on an ELISA reader at 450 nm (LABSYSTEM). These assays detected only human cytokines. The results are expressed as picograms per milliliter relating to a standardized dose curve of the relevant recombinant TNF-*α*.

### 2.5. Nitric Oxide (NO) Production

The stable nitrite (NO^−2^) concentration, being the end product of NO oxidation, was determined by the method described by Touil-Boukoffa and others (1998). Briefly, nitrite (NO^−2^)‏ was quantified by spectrophotometry (at 543 nm) in samples (plasma, supernatants of colonic mucosa culture) after reaction with Griess reagent. The NO^−2^ concentration was determined by extrapolation from a NaNO2 standard curve and expressed as *μ*M/mL [41].

### 2.6. RNA Extraction and Real-Time Quantitative PCR

Total RNA was extracted from colonic mucosa using a QIAGEN RNeasy kit (QIAGEN). After reverse transcription into cDNA with a Reverse Transcription Kit (Bio-Rad), qPCR was then performed on MyiQ single color RT-PCR detection system with SYBR Green Super Mix (Bio-Rad) and gene-specific primers were summarized in Table 1. We normalized gene expression amount to *β*-actin and GAPDH housekeeping gene and represented data as fold differences by the 2^−ΔΔCt^ method, where ΔCt = Ct target gene – Ct MG and ΔΔCt = ΔCt patients −  ΔCt control. Fold changes were calculated using the comparative Ct method.

### 2.7. Histological Analysis

Multiple colonic biopsies were taken from patients (active UC, *n* = 10; CAC, *n* = 9) and were fixed for 24 hours in buffered formaldehyde solution (10% in PBS) at room temperature. The biopsies were dehydrated by graded ethanol and embedded in paraffin (solidification point 60–62°C). Tissue sections (thickness 3 *μ*m) were deparaffinized with toluene, stained with hematoxylin and eosin (H&E). Digital images were captured with digital camera at ×400 resolution. Each colonic segment was evaluated based on the Geboes histology score. Scores can range from 0 to 5.4, with higher scores indicating more severe histological inflammation.

### 2.8. Immunohistochemistry

Sections (3 *μ*m) were cut from paraffin embedded tissues and mounted on positively charged super frost slides. Tissues were deparaffinized and rehydrated through graded alcohols. All sections were incubated in 3% hydrogen peroxide (10 min) to blockade endogenous peroxidase activity. Nonspecific binding was blocked by incubation (2 hours) in PBS containing 5% skim milk. The sections were subsequently incubated overnight at 4°C in monoclonal mouse anti-TLR4 (Invitrogen-Life Technologies, USA) (diluted 1 : 100 in phosphate buffered saline (PBS) containing 5% skim milk). Binding of the primary anti-TLR4 was detected with biotinylated rabbit anti-mouse immunoglobulin horse radish peroxidase- (HRP-) conjugated streptavidin (1 : 500). Immunoreactive complexes were detected using DAB system (Invitrogen-Life Technologies, USA). Slides were counterstained briefly in hematoxylin (Sigma Aldrich) and mounted in Eukit (Sigma Aldrich). Slides were observed using a standard microscope (Zeiss) and pictures were taken using a digital camera at ×100 resolution. The intensity of staining of each section was evaluated subjectively by three separate observers in 4 fields nonoverlapping using the following designations: 0–10% of cells stained, score 0; 11–25% of cells stained, score 1; 26–50% of cells stained, score 2; 51–100% of cells stained, score 3. Those scoring 0-1 were considered to be negative, and those scoring 2-3 were considered to be positive.

### 2.9. Western Blot

The biopsies were lysed with phosphate buffered containing 1% Triton X-100 and 1% protease inhibitor mixture (Sigma Aldrich). Protein concentrations were determined using a Bio-Rad Protein Assay (Bio-Rad, Marnes la Coquette, France) according to manufacturer's instructions. SDS-PAGE was performed according to the Laemmeli procedure using gradient precasts gels (4–12% gradient, Bis-Tris) (Invitrogen). Proteins were transferred on to PVDF membrane (Millipore, Molsheim, France) and probed with primary Ab (anti-TLR4, 1/1000) (Invitrogen-Life Technologies, USA) and the rabbit anti-*β*-actin as inner control. The membranes were incubated with peroxidase-conjugated secondary antibodies (anti-rabbit, 1 : 10000) (GE Healthcare, Wauwatosa, WI, USA) and washed again with blocking buffer. Specific protein signals were visualized using Western Lightning H Plus, ECL, Enhanced Chemiluminescence Substrate kit (PerkinElmer, Boston, MA, USA).

### 2.10. Immunohistofluorescence

Colonic mucosa sections (3 *μ*m) were saturated by incubation (2 hours) in PBS containing 5% skim milk and then permeabilised with 0.1% Triton X-100. A rabbit IgG1 monoclonal antibody (diluted 1/100), a mouse IgG1 monoclonal antibody (diluted 1/100), a rabbit IgG1 monoclonal antibody (diluted 1/100), and a rabbit IgG1 monoclonal antibody (diluted 1/50) were used as primary antibody for NF-*κ*B, Ikk, NOS2, and TNF-*α* detection, respectively. Fluorescein isothiocyanate- (FITC-) conjugated IgG was used as secondary antibody. Slides were covered with Kaiser's glycerin-PBS and observed using a standard microscope (Zeiss) and pictures were taken using a digital camera at ×100 resolution. The intensity of staining of each section was evaluated subjectively by three separate observers in 4 fields nonoverlapping using the following designations: 0–10% of cells stained, score 0; 11–25% of cells stained, score 1; 26–50% of cells stained, score 2; 51–100% of cells stained, score 3. Those scoring 0-1 were considered to be negative, and those scoring 2-3 were considered to be positive.

### 2.11. Statistical Analysis

All results were expressed as mean ± standard deviation. Data analysis was performed using Minitab 16. Student's *t*-test was used for comparison between different groups. Differences were considered to be statistically significant at *P* < 0.05.

## 3. Results

### 3.1. The Relationship between Nitric Oxide and TNF-*α* Levels in Plasma of UC and CAC Patients

In order to assess the involvement of TNF-*α* and NOS2 during UC and CAC, at first, we analyzed the NO and TNF-*α* production in vivo. Analysis of nitric oxide (NO) and circulating TNF-*α* production by Griess method and ELISA, respectively, revealed that NO and TNF-*α* levels are increased in all groups of patients in comparison to the healthy controls (*P* < 0.001) (Table 2). Interestingly, TNF-*α* levels are higher in sera of patients with CAC than in sera of patients with active UC (374.7 ± 16.31 pg/mL versus 291.48 ± 19.03 pg/mL) (Table 2). In contrast, no statistically significant difference in NO levels was observed between active UC and CAC patients (45.67 ± 6.14 *μ*M versus 43.91 ± 7.28 *μ*M) (*P* > 0.05) (Table 2).

**Figure 1:**
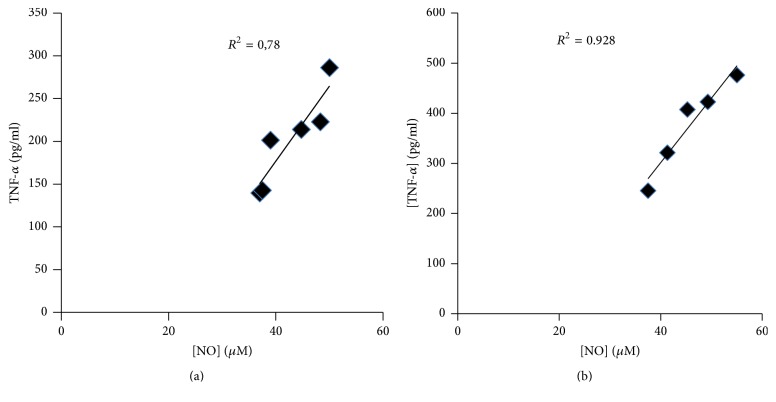
Correlation between the serum NO levels and the serum TNF-*α* levels in Algerian patients with (a) active UC and (b) CAC. (a) There is a significant correlation between NO levels and TNF-*α* levels in patients with active UC (*R*^2^ = 0.78). (b) There is a significant correlation between the NO levels and TNF-*α* (*R*^2^ = 0.92) levels in patients with CAC (TNF-*α*, tumor necrosis factor-alpha; NO, nitric oxide; UC, ulcerative colitis; CAC, colitis associated cancer).

As shown in Figure 1(a), there is a significant positive correlation between NO levels and TNF-*α* levels in plasma of active UC (*R*^2^ = 0.78; *P* < 0.01). A similar observation was observed in CAC patients, who presented linear positive correlation between NO levels and TNF-*α* (*R*^2^ = 0.92; *P* < 0.01) (Figure 1(b)).

### 3.2. TNF-*α* and NOS2 Expression in Colonic Mucosa of UC and CAC Patients: Correlation with Histological Damage

Considering the high levels of circulating NO and TNF-*α* assessed in vivo, we decided to determine the possibility of upregulation of NOS2 and TNF-*α*/TNFR expression in inflamed colonic mucosa of UC and CAC patients. Therefore, we analyzed the expression of messenger-RNAs (mRNAs) encoding TNF-*α* and TNF receptors (TNFRp55 and TNFRp75) in inflamed colonic mucosa from UC and CAC patients. The NOS2 (iNOS) mRNA expression was also analyzed by QPCR in these patients.

**Figure 2:**
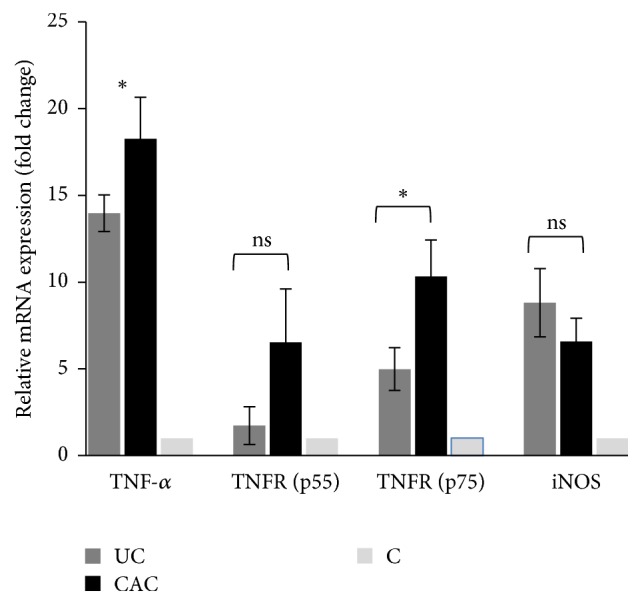
Messenger-RNA (mRNA) transcripts of TNF-*α*, TNFR (p55, p75), and iNOS were quantitated in colonic mucosa of patients with UC and CAC. Expression was normalized against *β*-actin and GAPDH housekeeping gene, and relative expression was represented data as fold differences by the 2^−ΔΔCt^ method, where ΔCt = Ct target gene − Ct MG and ΔΔCt = ΔCt inflamed colonic mucosa −  ΔCt normal colonic mucosa. Fold changes were calculated using the comparative Ct method. Data represent mean ± standard deviation. Significant difference between the two groups of patients (UC/CAC) is indicated (^*∗*^*P* < 0.05). ns, no significant difference, *P* > 0.05 (UC, ulcerative colitis, *n* = 12; CAC, colitis associated cancer, *n* = 5; C, control, *n* = 5).

As shown in Figure 2, analysis of TNF-*α*, TNF receptors, and NOS2 transcripts reveals that messenger-RNA transcript levels of the indicated genes are elevated in inflamed colonic mucosa from all groups of patients as compared with normal colonic mucosa. Moreover, TNF-*α* mRNA expressions are higher in colonic mucosa of patient's with CAC than in colonic mucosa of patients with active UC (*P* < 0.01). A similar observation was noted in CAC patients, who presented significantly higher TNFRp75 mRNA levels compared with active UC patients (~2-fold *P* < 0.01). In contrast, no statistically significant difference in TNFRp55 and NOS2 mRNA expression was detected between active UC and CAC patients (*P* > 0.05) (Figure 2).

**Figure 3:**
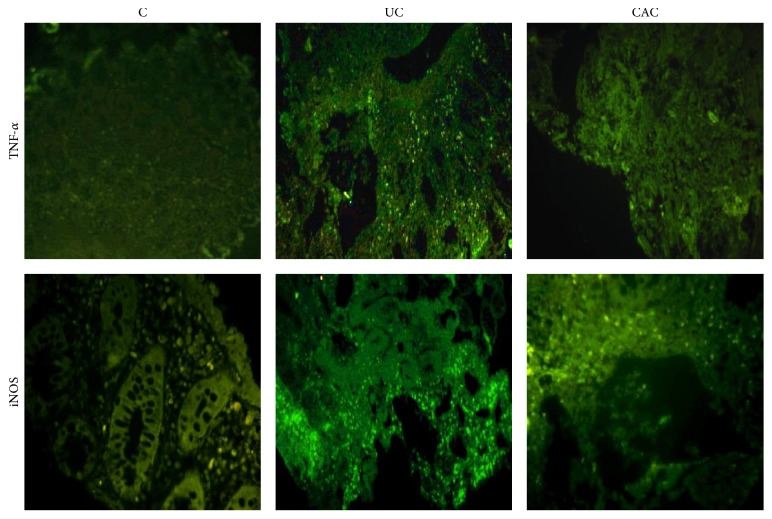
TNF-*α* and NOS2 expression in inflamed colonic mucosa of active UC and CAC patients detected by immunofluorescent staining (IF). TNF-*α* and NOS2 expression was upregulated in inflamed colonic mucosa (higher inflammation score) of active UC and CAC patients compared with control. Immunofluorescence staining of NOS2 (score 3) and TNF-*α* (score 3) was more intense in inflamed colonic mucosa of UC patients compared with control (score 0). High score and intensity of NOS2 (score 3) and TNF-*α* (score 3) were also evaluated in colonic mucosa of CAC patients (UC, ulcerative colitis, *n* = 5; CAC, colitis associated cancer, *n* = 3; C, control, *n* = 4).

In addition, immunofluorescence analysis showed that TNF-*α* protein expression was upregulated in inflamed colonic mucosa of all cohorts of patients in comparison with the controls (Figure 3). We also noted that NOS2 expression was upregulated in colonic mucosa of UC and CAC patients compared with the controls (Figure 3).

**Figure 4:**
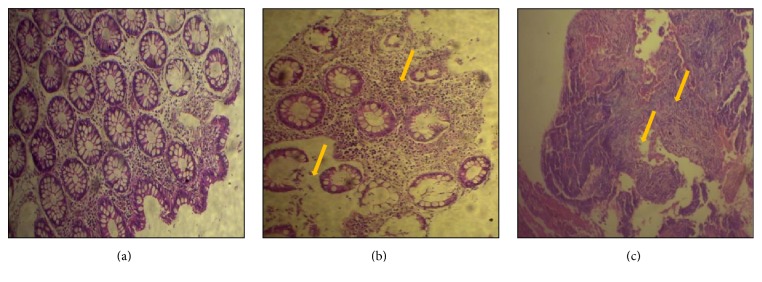
Representative photomicrographs of H&E-stained (a) colonic mucosa from control, (b) active UC patients (severe inflammation; higher score 5.4), and (c) CAC patients (severe inflammation; higher score 5.4). Arrows show cellular infiltrate and crypt destruction (UC, ulcerative colitis, *n* = 10; CAC, colitis associated cancer, *n* = 9; C, control, *n* = 5).

The histological study indicated a profound colonic inflammation (higher score) characterized by crypt destruction. Inflammatory cells infiltration into mucosa in UC patients (Figure 4(b)) was also noticed compared to colonic mucosa control showing normal structure (Figure 4(a)). A similar observation was noticed in colonic mucosa of CAC patients (Figure 4(c)).

Considering the overexpression of iNOS and TNF-*α* in inflamed colonic mucosa of UC and CAC patients, we also decided to evaluate the percentage of mixed leukocytes (inflammatory cell infiltrate) in areas with higher or low expression of iNOS and TNF-*α*.

**Figure 5:**
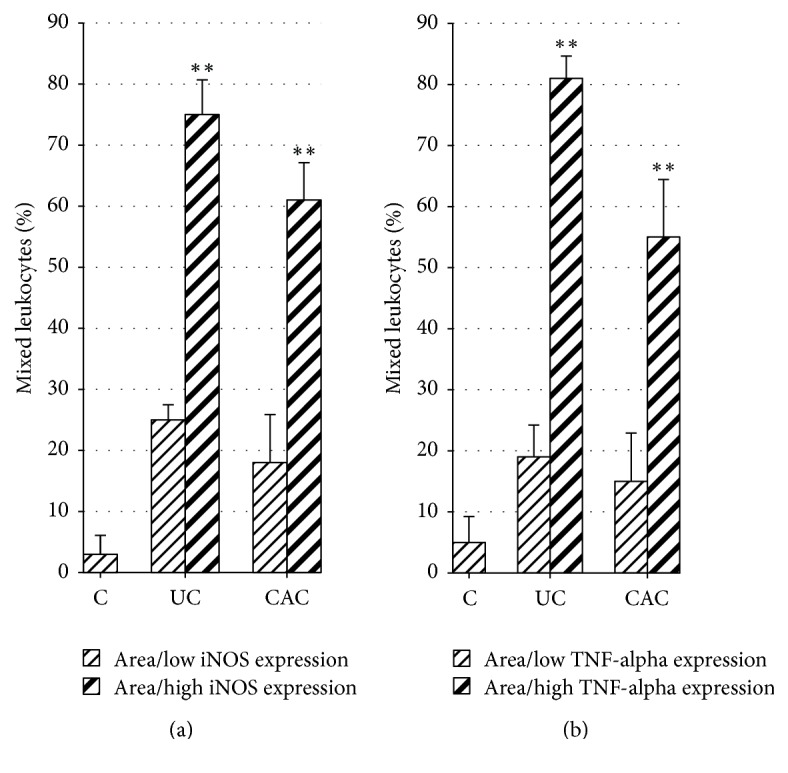
Percentage of mixed leukocytes in areas with high or low expression of iNOS and TNF-*α*. Mixed leukocytes (inflammatory cell infiltrate) were counted in 4 wells nonoverlapping in areas of iNOS/TNF-*α* overexpression and areas with low expression. High density of mixed leukocytes was observed in areas of iNOS and TNF-*α* overexpression as compared with areas of low iNOS/TNF-*α* expression in colonic mucosa of UC and CAC patients (^*∗∗*^*P* < 0.01).

Interestingly, the percentage of mixed leukocytes is higher in areas of iNOS overexpression than in areas with low iNOS expression in UC and CAC patients (Figure 5(a); UC: 75% versus 25%; CAC: 61% versus 18%) (*P* < 0.01). A similar observation was noted in areas with higher expression of TNF-*α* when compared with areas of low TNF-*α* expression (Figure 5(b); UC: 81% versus 19%; CAC: 55% versus 15%) (*P* < 0.01). Our finding showed that the overexpression of iNOS (NOS2) and TNF-*α* in inflamed colonic mucosa was associated with histological damage.

### 3.3. LPS/Toll Like Receptor-4 (TLR4) Signaling Induced Nitric Oxide and TNF-*α* Production by Colonic Mucosa through NF-*κ*B Pathway

Several studies have demonstrated that TLR4 expression is known to be low in the normal colon but increased in inflammatory bowel disease (ulcerative colitis and Crohn's disease) [16, 26, 28]. We hypothesized that TLR4 expression is upregulated in colitis associated tumors and plays a pivotal role to induce iNOS and TNF-*α* expression through NF-*κ*B pathway.

Colon specimens from patients with UC or CAC were examined for TLR4 mRNA expression by Q-PCR. We also evaluated TLR4 protein expression in inflamed colonic mucosa by Western blot analysis and immunohistochemical staining. In addition, NF-*κ*B and iKK protein expression was assessed in inflamed colonic mucosa from the same patients by immunofluorescent staining.

**Figure 6:**
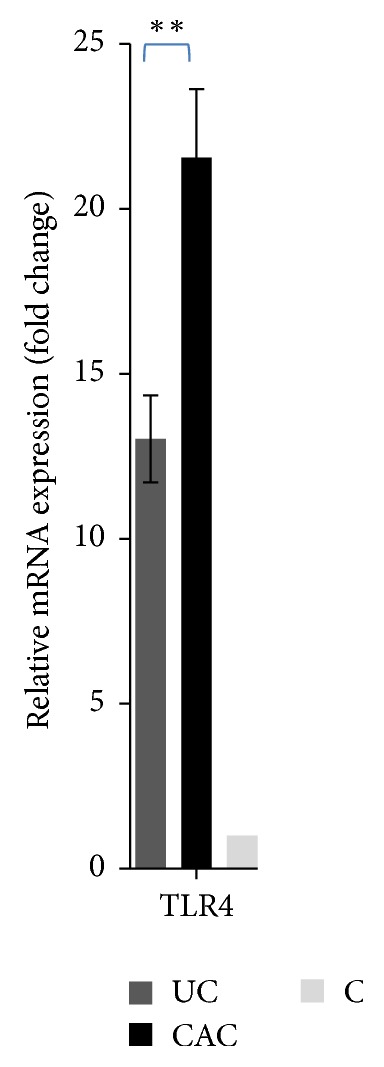
mRNA transcripts of TLR4 were quantitated in colonic mucosa of UC and CAC patients. Expression was normalized against *β*-actin, HPRT, and GAPDH housekeeping gene, and relative expression was represented data as fold differences by the 2^−ΔΔCt^ method, where ΔCt = Ct target gene − Ct MG and ΔΔCt = ΔCt inflamed colonic mucosa −  ΔCt normal colonic mucosa. Fold changes were calculated using the comparative Ct method. Data represent mean ± standard deviation. Significant difference between patients (UC/CAC) and controls is indicated (*P* < 0.001). Significant difference between 2 groups of patients (UC/CAC) is indicated (^*∗∗*^*P* < 0.01) (UC, ulcerative colitis, *n* = 12; CAC, colitis associated cancer, *n* = 5; C, control, *n* = 5).

Transcriptomic analysis of TLR4 showed that levels of mRNA transcripts of the indicated gene are elevated in inflamed colonic mucosa of all cohorts of patients in comparison with the controls (*P* < 0.001), especially in CAC patients (*P* < 0.01) (Figure 6).

**Figure 7:**
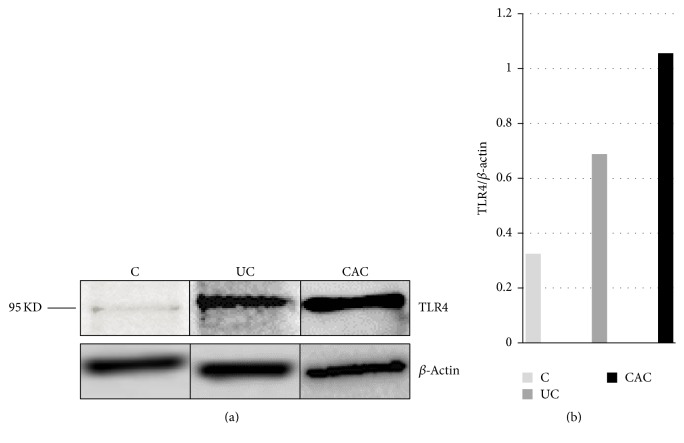
The expressions of TLR4 in colonic mucosa of UC and CAC patients detected by Western blot. (a) Representative Western blot analysis of total biopsies homogenate from colonic mucosa of active UC and CAC patients. A TLR4 specific polyclonal antibody recognized a protein whose molecular weight was approximately 95 kDa. (b) The relative intensity of TLR4 (C: control, colonic mucosa, *n* = 3; UC: ulcerative colitis, *n* = 5; CAC: colitis associated cancer, *n* = 3).

**Figure 8:**
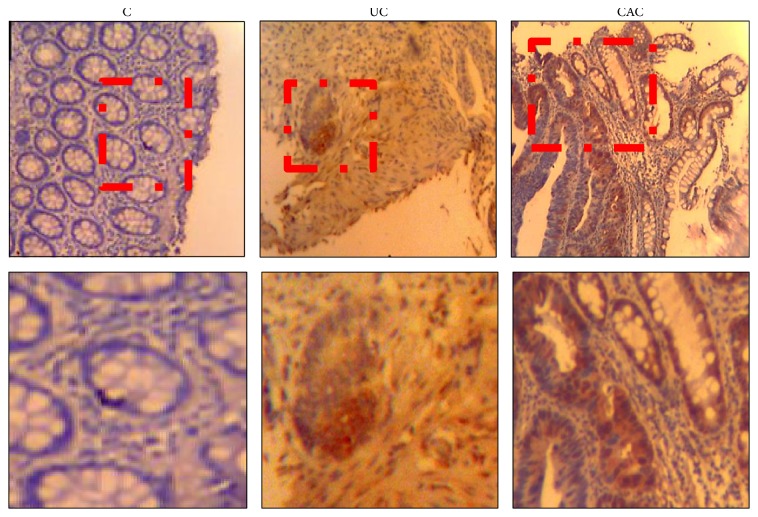
Immunohistochemical expression of TLR4. Control colonic mucosa shows a low expression of TLR4 (score 0). This expression was profoundly increased in colonic mucosa of patients UC (score 3) and CAC (score 3) (UC: ulcerative colitis, *n* = 6; CAC: colitis associated cancer, *n* = 4; C: control, normal mucosa, *n* = 3).

In our current study, we also observed with interest that TLR4 protein expression was upregulated in colonic mucosa of patients compared with the controls, especially in patients with CAC (Figures 7 and 8).

**Figure 9:**
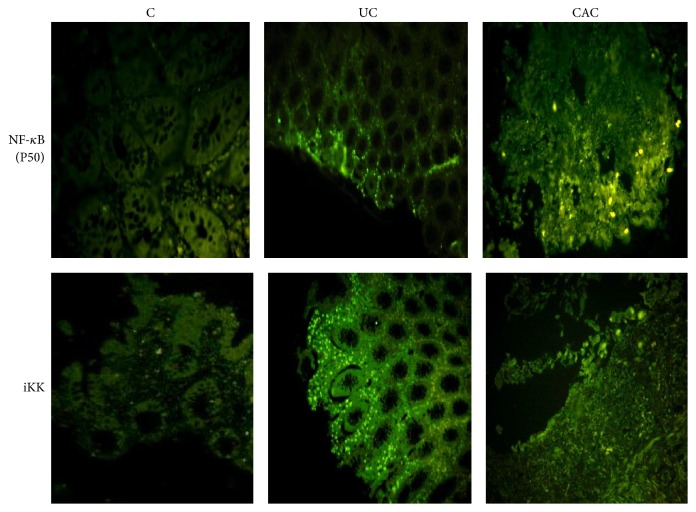
The expression of NF-*κ*B (P50) and ikk*γ* in colonic mucosa of UC and CAC patients detected by IF. NF-*κ*B (score 3) and ikk*γ* (score 3) expression was upregulated in colonic mucosa of active UC and CAC patients compared with control (score 0) (UC: ulcerative colitis, *n* = 6; CAC: colitis associated cancer, *n* = 4; C: control, normal mucosa, *n* = 3).

Immunofluorescent staining analysis indicated that NF-*κ*B and iKK protein expression is highly increased in colonic mucosa of UC patients in comparison with controls (Figure 9). A similar result was reported in CAC patients, who presented higher NF-*κ*B/iKK expression compared with controls (Figure 9).

To further confirm the relationship between LPS/TLR4 and TNF*α*/NOS2 induction, as well as the role of NF-*κ*B signaling in ulcerative colitis and colitis associated tumorigenesis, we used ex vivo experiments. Colonic mucosa of UC and CAC patients was stimulated with LPS (10 *μ*g/mL) in the absence or presence of SN50 (50 *μ*M/mL). NO and TNF-*α* production was analyzed after 24 hours of incubation.

**Figure 10:**
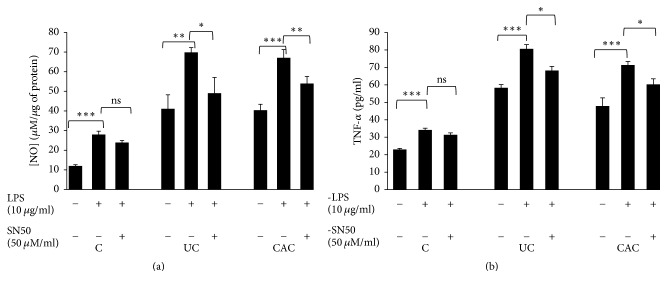
Role of NF-*κ*B signaling on nitric oxide (a) and TNF-*α* (b) production by colonic mucosa stimulated with LPS (10 *μ*g/ml) in UC and CAC patients. Values represent mean ± standard deviation. Significance compared with control: ^*∗∗∗*^*P* < 0.001, ^*∗∗*^*P* < 0.01, and ^*∗*^*P* < 0.05; ns: no significant difference (UC: ulcerative colitis; CAC: colitis associated cancer; C: control, normal mucosa).

As shown in Figure 10, stimulation with LPS increases NO and TNF-*α* production by inflamed colonic mucosa from patients with active UC compared with unstimulated biopsies (*P* < 0.01). These findings were also observed in the culture with colonic mucosa of CAC patients (*P* < 0.001) (Figure 10). Our study showed that SN50 (50 *μ*M/mL) inhibited LPS induction of NO and TNF-*α* production in inflamed colonic mucosa cultures in all groups of patients (Figure 10). The SN50 in colonic mucosa culture stimulated with LPS from CAC patients causes a significant decrease of the amount of NO (−1.24-fold, *P* < 0.01) and TNF-*α* (−1.18-fold, *P* < 0.05) compared with colonic mucosa stimulated with LPS alone (Figure 10). The same profile was observed for colonic mucosa treated with SN50 from the patients with UC (*P* < 0.05) (Figure 10).

### 3.4. All-Trans Retinoic Acid (AtRA) Modulates a TNF-*α* Major Inflammation Cytokine and NO Synthase 2 (iNOS) in Inflamed Colonic Mucosa of UC and CAC Patients

Having demonstrated the link between TLR4/NF-*κ*B and TNF-*α*/NOS2 induction in colorectal tumors, we focused our attention to elucidate the immunomodulatory effect of all-trans retinoic acid in NOS2 and TNF-*α* expression in inflamed colonic mucosa cultures. We used RT-PCR to study NOS2 and TNF-*α* mRNAs in colonic mucosa pretreated with AtRA (10^−7 ^M) and stimulated with LPS. We also evaluated NO and TNF-*α* production by these colonic mucosae using Griess method and ELISA, respectively. Finally, we examined the ability of AtRA to modulate NOS2 protein expression in colonic mucosa from UC and CAC patients in response to LPS.

**Figure 11:**
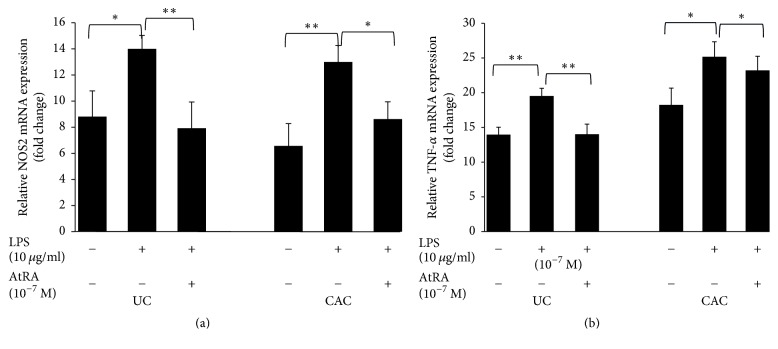
Effect of LPS (10 *µ*g/ml) in the presence or absence of AtRA (10^-7 ^M) on NO2 (a) and TNF-*α* (b) mRNA expression in colonic mucosa of patients with active UC and CAC. Values represent mean ± standard deviation. Significance compared with control: ^*∗*^*P* < 0.05, ^*∗∗*^*P* < 0.01 (UC: ulcerative colitis; CAC: colitis associated cancer).

**Figure 12:**
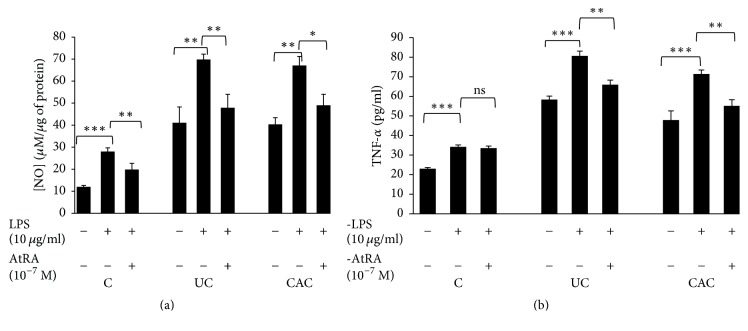
Effect of LPS (10 *µ*g/ml) in the presence or absence of AtRA (10^-7 ^M) on NO (a) and TNF-*α* (b) production by colonic mucosa of patients with active UC and CAC. Values represent mean ± standard deviation. Significance compared with control: ^*∗*^*P* < 0.05, ^*∗∗*^*P* < 0.01, and ^*∗∗∗*^*P* < 0.001. ns, no significant difference, *P* > 0.05 (UC: ulcerative colitis; CAC: colitis associated cancer; C: control, normal mucosa).

Our study showed that AtRA inhibited LPS induction of NOS2 and TNF-*α* mRNA expression in inflamed colonic mucosa cultures of all patients (Figure 11). Moreover, the pretreatment of colonic mucosa of CAC patients with AtRA decreased significantly NOS2 (−1.50-fold, *P* < 0.05) and TNF-*α* (−1.25-fold, *P* < 0.05) mRNA expression compared with colonic mucosa stimulated with LPS alone (Figures 11(a) and 11(b), resp.). The same profile was reported for colonic mucosa treated with AtRA from the patients with UC (*P* < 0.01) (Figures 11(a) and 11(b)).  Figure 12 illustrates the significant downregulation of NO and TNF-*α* production by UC and CAC patients' colonic mucosa pretreated with AtRA in response to LPS compared with the controls (*P* < 0.001).

**Figure 13:**
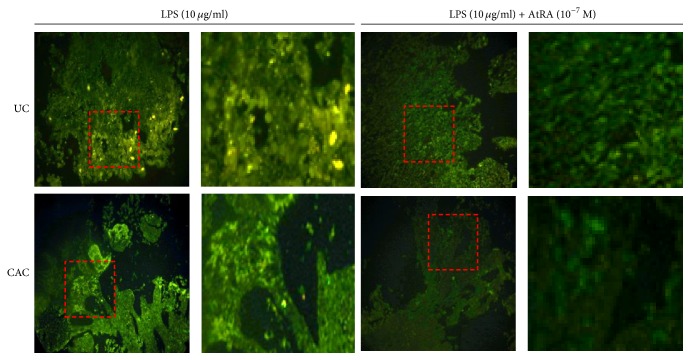
Effect of LPS (10 *µ*g/ml) in the presence or absence of AtRA (10^-7 ^M) on NOS2 (iNOS) expression in inflamed colonic mucosa of patients with active UC and CAC. AtRA reduced NOS2 expression in colonic mucosa (UC: ulcerative colitis; CAC: colitis associated cancer).

Finally, we have investigated whether AtRA inhibits NO production directly by modulating iNOS expression. Interestingly, we report in our work that the pretreatment with AtRA decreased significantly NOS2 expression in colonic mucosa stimulated with LPS in all groups of patients (Figure 13). Indeed, the immunofluorescence analysis showed that NOS2 expression was downregulated in colonic mucosa cultures pretreated with AtRA in the presence of LPS in all groups of patients (UC and CAC) compared with controls (Figure 13).

## 4. Discussion

It is possible that overproduction of inflammatory mediators, including proinflammatory cytokines, during IBD may facilitate the development and progression of colorectal cancer [8, 42–44]. It was reported that several key genes in the inflammatory process such as NF-*κ*B provide a mechanistic link between inflammation and cancer [45–48]. Activation of TLRs and TNFR leads to NF-*κ*B activation and induced the expression of many proinflammatory molecules including NO synthase 2 (iNOS) [49, 50]. A growing number of studies have reported the key role of TLR4/NF-*κ*B/TNF-*α* signaling to promote tumor growth in experimental models of colitis associated cancer [44, 51–53], whereas data from human CAC are rare.

In our study, we observe with interest the positive correlation between TNF-*α* and NO in plasma of patients with UC and CAC. Our findings suggest that TNF-*α* is involved in upregulation of NOS2 induction in patients with UC and CAC. We also evaluated the expression of mRNAs of TNF-*α*, TNFR (p55, p75), and NOS2 (iNOS) in colonic mucosa of patients with UC and CAC. Interestingly, we have noticed significant levels of mRNA transcripts of the indicated genes in all groups of patients. In addition, immunofluorescence analysis showed that TNF-*α* and NOS2 expression was also upregulated in colonic mucosa of all cohorts of patients in comparison with the controls. Our findings are in agreement with previous data showing the overexpression of TNF-*α* [54] and NOS2 [11] in the inflamed intestinal mucosa in UC patients, human colon carcinoma tissue [55], and murine model of carcinoma arising on colitis [56]. Both TNF-*α* and nitric oxide have been suggested to be an important mediators involved in the initiation of intestinal inflammation and perpetuation of local tissue damage [21, 46]. Popivanova et al. showed that TNF-*α* is a crucial mediator of the initiation and progression of colitis associated colon carcinogenesis [51]. Furthermore, in several models of chronic inflammation-associated carcinogenesis, TNFR was predominantly expressed by leukocytes infiltrating the lamina propria and submucosal regions of the colon [57]. The TNF-TNFR axis probably contributes to the development of chronic inflammation-associated colon carcinogenesis process. The absence of TNFR1 reduced the infiltration of macrophages and neutrophils, a major source of COX-2, and eventually depressed colon carcinogenesis [58]. The excess prostaglandin E2 generated by COX2 in the recovery period of colitis can induce neovascularization, aberrant epithelial cell proliferation, and activation of the Wnt/*β*-catenin pathway, resulting in the development and growth of colitis associated neoplasms [59, 60].

Previous works indicated that inducible nitric oxide synthase (iNOS) is expressed by several human gastrointestinal neoplasms including gastric cancer [61], colonic adenomas [62], Barrett's esophagus, and associated adenocarcinomas [63]. These data suggest that iNOS may play a pivotal role in the initiation and promotion and probably progression of cancers arising within a background of inflammation [64, 65]. However, the mechanisms by which inflammation stimulates the development of cancer remain elusive and are expected to vary from colitis associated CRC to other forms of CRC [42].

Our results show that histologic examination of biopsies obtained from CAC patients arising in several ulcerative colitis reveals a severe mucosal degeneration, crypt loss, and destruction of epithelium in addition to cellular infiltrates. These observations correlate with the high NOS2 and TNF-*α* expression in colonic mucosa of the same patients. It can be argued that NO are clearly implicated in the mucosal injury observed during CAC pathogenesis.

In our study, we also demonstrate that TLR4 transcript is highly expressed in inflamed colonic mucosa of UC patients and CRC patients arising in chronic ulcerative colitis. In addition, our Western blotting and immunohistochemistry analysis showed the upregulation of TLR4 in colonic mucosa of UC and CAC patients. This finding is in agreement with previous data showing that patients with active UC had significantly more TLR4-positive epithelial cells than controls [66, 67]. In the same context, several studies reported an increase in the TLR4 expression in colon cancer cell lines (HT29, SW480, and KM20) [68, 69]. Fukata et al. showed that TLR4 is overexpressed in CAC tissue of UC patients [70]. Furthermore, several preclinical studies showed that mice deficient in TLR4 are markedly protected against colitis associated neoplasia [66, 71].

Collectively, these data raise a clear link between TLR4 and CAC development. TLR4 triggers elevated production of prostaglandin E2, influences epidermal growth factor receptor signaling (EGFR), and increases TNF-*α*/NOS2 induction in chronic colitis [71].

LPS has been shown to bind directly to the TLR4/MD2 receptor complex that initiates the intracellular signaling cascade in a MyD88-dependent or MyD88-independent manner [72, 73]. LPS-induced TLR4 signaling leads to activation of various downstream pathways including NF-kB [74]. In our present study, we evaluated the expression of NF-kB and iKK in inflamed colonic mucosa from UC and CAC patients. Our finding showed that NF-*κ*B and iKK protein expression is highly increased in colonic mucosa in all groups of patients. In fact, some studies have shown the overexpression of NF-*κ*B in the inflamed intestinal mucosa in UC and CAC patients and murine model of CAC [44, 75].

In our study, the effects of NF-*κ*B activation or inhibition on TNF*α*/NOS2 induction are confirmed by ex vivo experiments. Our results showed that SN50 inhibited LPS induction of NO and TNF-*α* production in inflamed colonic mucosa cultures in all groups of patients. Although SN50 peptide could also competitively inhibit many transcription factors such as STAT and AP-1, entering nucleus, it mainly and firstly inhibits the NF-*κ*B pathway after LPS activation.

The pivotal role of NF-kB signaling in tumor progression was provided in the AOM-DSS model for colitis associated colorectal cancer by Greten et al. [76], which showed that deletion of Ikk in intestinal epithelial cells resulted in decreased numbers of tumors. In the same way, another team had also recently demonstrated that TNF-*α* inhibition in the animal model prevents the development of CAC via blockade of TNF receptor 1 (TNFR1) signaling in infiltrating hematopoietic cells such as neutrophils and macrophages [52]. TNF-*α* potently induces NF-*κ*B activation but can also be a NF-*κ*B-target gene [52, 77]. These data suggest that TLR4/TNF-*α* through NF-*κ*B activation could influence CAC development.

Several hematopoietic and nonhematopoietic lineages within the gastrointestinal tract, including macrophages, dendritic cells, and lymphocytes T share the capacity to synthesize retinoic acid [78, 79]. A number of studies demonstrated that AtRA has an important modulating role in innate immunity, with the most recent reports showing that RA has a central function in the differentiation of dendritic cells (DCs), the key APCs for activating naive T cells [80, 81]. Colorectal tumor cells appear to lose the ability to produce AtRA [82] while, at the same time, they appear to increase AtRA degradation via the cytochrome P450 enzyme (CYP26A1), a major retinoic acid catabolic enzyme [83, 84].

The biological effects of AtRA are mainly mediated by two families of nuclear retinoic acid receptors (RARs), each consisting of three receptor subtypes designated by *α*, *β*, and *γ*: the RARs and the retinoid X receptors (RXRs) that are expressed in lymphoid cells and act as transcription factors to regulate cell signaling, differentiation, and tumor suppression [85]. In addition to the higher CYP26A1 expression and the consequential AtRA resistance, as CRC progresses, RAR *β* was downregulated in mice bearing mutations in the Apc tumor suppressor gene (ApcMIN mouse), human FAP adenomas, and human sporadic colon carcinomas [86, 87]. The downregulation of RAR *β* may lead to alteration of cell growth and differentiation in the colon and rectum, thus contributing to the progression of colorectal cancer. Several series of preclinical models of colon carcinogenesis and in vitro system of a retinoid sensitive/resistant human colon carcinoma cells lines suggested that retinoid mediated autoinduction of the endogenous RAR *β* gene may play a crucial role in mediating the biological effects of retinoids [88, 89]. A study of Møllersen et al. using AtRA in the ApcMIN mouse found enhanced adenoma formation within the small intestine. In contrast, no changes in adenoma formation were observed within colon of ApcMIN mice after administration of AtRA [90]. However, the complexities of retinoic acid responses in the intestine require further investigation and greater understanding to propose new therapies, such as investigating the implication of these factors in the retinoid synthesis or in their mechanisms of action during inflammation. In this context, we propose to investigate the efficacy of AtRA as a new strategy in order to improve the current therapy in colorectal cancer, including CAC.

In this sense, we aimed to explore the mechanisms by which AtRA regulates TNF-*α* and iNOS expression in colonic mucosa cultures stimulated with LPS. Our current studies demonstrated that AtRA decreased TNF and NOS mRNA expression in inflamed colonic mucosa stimulated with LPS and also inhibited TNF-*α* and NO production by some colonic mucosa. Our finding suggests that AtRA modulates LPS-TLR4 signaling targeting TNF-*α* and nitric oxide synthase 2 expression in UC and CAC. These results are in agreement with previous studies investigating the inhibitory effect of AtRA on NOS2 expression in human colon cancer cell lines [91–93].

Several studies reported that AtRA could downregulate NOS2 gene expression by direct mechanisms mediated by RARa binding to the putative RARE in the promoter or the NOS2 gene [94]. AtRA could also regulate directly this expression by a protein-protein interaction with some transcription factors such as AP-1 [95] and/or NF-kB [96, 97], both reported to be involved in the induction of gene expression of previous inflammatory mediators, including NOS2 and TNF-*α* [52]. In addition, several data suggest that inhibition of NF-kB translocation to the nucleus may contribute to the anti-inflammatory mechanisms of AtRA [98, 99]. Consistent with several findings from other groups [100–102], our results showed that the immunomodulatory effects of AtRA on TNF-*α* production could be the result of direct inhibition of cytokine gene expression and could therefore indirectly decrease NOS2 gene expression.

Overall, our present study strongly supports (i) the pivotal role of TLR4/NF-*κ*B signaling in UC and CAC pathogenesis through NO synthase 2 (NOS2) and TNF-*α* induction and (ii) AtRA downregulating NO synthase 2 and TNF-*α* expression targeting the LPS/TLR4/NF-*κ*B pathway. Therefore, we suggest that AtRA has a potential value in new strategies to improve the current therapy and probably in the clinical prevention of CAC development and progression.

## Figures and Tables

**Table 1:** Summary of primers sequences used for qRT-PCR.

Gene	5′ primer (5′-3′)	3′ primer (5′-3′)
*TNF-α*	ATCTTCTCGAACCCCGAGTGA	GGAGCTGCCCCTCAGCTT
*TNFRp55*	GCTTCAGAAAACCACCTCAGACA	CCGGTCCACTGTGCAA
*TNFRp75*	CAACACGACTTCATCCACGG	GACGTGCAGACTGCATCCAT
*TLR4*	CCCGACAACCTCCCCTTCT	TGCCCCATCTTCAATTGTCTG
*NOS2*	TGACCCTGAGCTCTTCGAAATC	AGGGCGTACCACTTTAGCTCC

TNF-*α*: tumor necrosis factor-alpha; TNFRp55/p75: tumor necrosis factor receptor; TLR4: toll like receptor-4; NOS2 (iNOS): NO synthase 2; NO: nitric oxide.

**Table 2:** Sera TNF-*α* and NO concentrations in patients with ulcerative colitis and colitis associated cancer.

	[TNF-*α*] (pg/ml)	NO (*μ*M)
UC (*n* = 35)	291,48 ± 19,03 (*P*< 0,001)	45,67 ± 6,14 (*P*< 0,001)
CAC (*n* = 9)	374,7 ± 16,42 (*P*< 0,001)	43,91 ± 7,28 (*P*< 0,001)
HD (*n* = 18)	18,54 ± 5,31	21,32 ± 5,16

Values represent the mean ± standard deviation.

Significance compared with healthy donors: *P* < 0.001.

TNF-*α*: significant difference between the two groups of patients (active UC/CAC) is indicated (*P* < 0.05).

NO: no significant difference between the two groups of patients (UC/CAC) is indicated (*P* > 0.05).

HD, healthy donors; UC, ulcerative colitis; CAC, colitis associated cancer.
